# Editorial: Apoptotic Cell Clearance in Health and Disease

**DOI:** 10.3389/fimmu.2018.02154

**Published:** 2018-10-02

**Authors:** Estee Kurant, Amiram Ariel, Kirsten Lauber

**Affiliations:** ^1^Departments of Biology and Human Biology, Faculty of Natural Sciences, University of Haifa, Haifa, Israel; ^2^Department of Radiation Oncology, University Hospital, Ludwig-Maximilians-University, Munich, Germany

**Keywords:** phagocytes, apoptosis, engulfment of debris, tissue maintenance, immuneregulation, efferocytosis

Clearance of apoptotic cells is essential for proper development, homeostasis, and termination of immune responses in multicellular organisms. Thus, the cellular and molecular players orchestrating the sequential events of this process are of great scientific interest. Research in the last 20 years has revealed that specific ligand-receptor axes mediate the attraction of immune cells toward apoptotic targets and the interactions between apoptotic cells and professional as well as non-professional phagocytes that engulf them. Moreover, phagocytosis of apoptotic cells (also known as efferocytosis) has been shown to induce significant phenotypic changes in the engulfing cell implicating that it is a major fate-determining event for phagocytes. Efferocytosis is of pivotal importance for the resolution of inflammation as well as for embryonic development and tissue morphogenesis. Accordingly, defects in dying cell clearance can strongly impair the mentioned processes and thus can result in severe health threats, including chronic inflammation, autoimmunity, atherosclerosis, bone loss, obesity, infertility, neurodegeneration, fibrosis, and cancer.

This volume compiles 24 manuscripts covering various aspects of apoptotic cell removal during normal development and homeostasis as well as during tumorigenesis and regenerative processes following injury. Eight of the manuscripts present original research on molecular mechanisms underlying the emergence and function of professional and non-professional phagocytes emphasizing the critical elements required for efficient clearance of apoptotic and necrotic cells. The remaining 16 papers provide overviews of evolutionarily conserved basic mechanisms and distinct efferocytosis pathways operating in particular organs and tissues under normal and/or pathological conditions, such as cancer, autoimmune diseases, injury, and viral infection.

Using *Drosophila melanogaster* embryonic macrophages as a model system for development of professional phagocytes Shlyakhover et al. demonstrate that the GATA transcription factor Serpent is critical for the specific expression of the phagocytic receptors Six-Microns-Under (SIMU), Draper (Drpr), and Croquemort (Crq). The authors show that each of these receptors is essential for the establishment of efferocytic ability, thereby unraveling crucial molecular aspects of *Drosophila* macrophage development.

Two research articles are dedicated toward the tyrosine kinase receptors TYRO3, AXL, and MERTK (collectively named TAM receptors), which are fundamental regulators of inflammatory responses and efferocytosis. Lumbroso et al. report increased expression and release of Protein S (PROS1), a known ligand for all TAM receptors, in macrophages during the resolution of inflammation. In turn, PROS1 acts as a key effector molecule in regulating efferocytosis as well as maturation and reprogramming of macrophages, identifying it as a new potential therapeutic target in the context of inflammatory and fibrotic disorders. Geng et al. provide evidence that activation of TAM receptors by their known ligand GAS6 requires γ-carboxylation of its N-terminal Gla domain and complex formation with phosphatidylserine (PS), generating a functional protein/lipid ligand for TAMs.

Two further research papers address the impact of known efferocytic mediators on the clearance of apoptotic or necrotic cells. The study by Tacnet-Delorme et al. provides first experimental evidence for a direct interaction between complement C1q and the neutrophil-specific serine protease Proteinase 3 which is externalized together with PS on apoptotic cells. This interaction impairs C1q-dependent efferocytosis, suggesting implications of this mechanism for the resolution of inflammation and/or autoimmunity. Grossmayer et al. report increased levels of lysophosphatidylcholine (LPC, a dying cell-derived “find-me” signal) in the sera of systemic lupus erythematosus (SLE) patients, which inhibit the clearance of dead cells by macrophages *in vitro*. The authors suggest that high levels of LPC may interfere with macrophage chemotaxis toward their dead cell targets, thus contributing to the establishment and/or maintenance of SLE disease.

Three research articles focus on microenvironmental aspects of apoptotic cell clearance. Graubardt et al. uncover a new role of Ly6C^hi^ monocytes and macrophages derived thereof (MoMF) in regulating neutrophil activity and clearance during the resolution of acetaminophen-induced liver injury (AILI). Initially, liver-infiltrating Ly6C^hi^ monocytes regulate innate immune functions and survival of neutrophils following injury, while later on their MoMF descendants exert clearance of apoptotic neutrophils during the resolution phase. Yang et al. describe a novel mechanism underlying the pro-coagulant activity of apoptotic cells through coagulation factor XII, which preferentially binds to apoptotic cells via PS and becomes activated, thus initiating an intrinsic coagulation pathway. Michaeli et al. demonstrate that *ex vivo* generated pro-resolving CD11b^low^ macrophages (Mres) secrete anti-angiogenic mediators, including endostatin, thereby inhibiting angiogenesis *in vitro* and *in vivo*. Apparently, this macrophage phenotype plays an important role in terminating tissue repair and restoring tissue structure.

The remaining articles include three mini reviews and thirteen reviews. Four of them discuss molecular aspects of the multi-step process of efferocytosis, including interactions of various “eat-me” signals with their cognate phagocytic receptors as well as consequences for anti-inflammatory and regenerative responses. The mini review by Barth et al. focuses on the molecular details of the “phagocytic synapse” which facilitates phagocytosis and subsequent signaling events, such as surface alterations and molecular opsonization. Hughes et al. discuss how phagocytes manage to respond appropriately to apoptotic cells in different immunological settings during everyday tissue turnover, tissue damage, infection, and/or inflammation.

Along similar lines, Gordon and Pluddemann accentuate the wide spectrum of phagocytic responses upon efferocytosis emanating from the variety of targets and effector cells. Zheng et al. summarize the current knowledge of apoptotic cell clearance in *D. melanogaster*.

Three reviews emphasize the role of non-professional phagocytes in the context of organ-specific efferocytosis. Davies et al. discuss recent research on efferocytosis by epithelial cells in the liver. Serizier and McCall survey phagocytosis of apoptotic germline cells by follicular epithelial cells in the *D. melanogaster* ovary with comparison to similar mechanisms in *Caenorhabditis elegans* and mammals. DeBerge et al. cover emerging knowledge on efferocytosis in the heart, including its role in cardiac development, homeostasis, and disease.

Six reviews focus on the impact of apoptotic cells on their cellular microenvironment with regards to immune homeostasis, treatment of autoimmunity, and anti-viral responses. Dalli and Serhan review the role of microvesicles and apoptotic cells in the production of specialized pro-resolving mediators as well as the biological actions of the latter during efferocytosis. Szondy et al. focus on anti-inflammatory mechanisms triggered by apoptotic cells during their removal. Trahtemberg and Mevorach summarize signaling events induced by apoptotic cells in macrophages and dendritic cells that direct immune-silencing and tolerance. The authors also discuss the use of apoptotic cells as therapeutic agents in mice and humans. Saas et al. provide an overview of the mechanisms behind this approach and suggest how it may be utilized to treat autoimmune arthritis. Manfredi et al. focus on the events that determine neutrophil fate amid phagocytosis and formation of neutrophil extracellular traps (NET) and their potential exploitation for the development of novel therapeutic approaches. Nainu et al. highlight an evolutionarily conserved anti-viral response that relays on apoptosis-dependent phagocytosis of virus-infected cells.

Three additional review articles discuss the role of apoptotic cells as important effectors in an oncological context of the tumor microenvironment. Ucker and Levine describe how tumor cells hijack conserved homeostatic processes instigated by apoptotic cells, including wound healing and regenerative processes, in order to promote cancer development and progression. Lynch et al. discuss how extracellular vesicles (EVs) derived from apoptotic tumor cells mediate host responsiveness to cell death in cancer and suggest that the monitoring of such EVs and their cargoes will improve cancer diagnosis, staging, and therapeutic efficacy. Jung et al. survey iron handling in tumor-associated macrophages highlighting the effect of dying tumor cells on an iron-release macrophage phenotype which appears to affect tumor progression.

Altogether, the articles in this volume cover a wide spectrum of aspects in apoptotic cell clearance and illuminate its high degree of complexity. A more detailed understanding of the molecular and cellular mechanisms governing this eminent process will enable us to unravel potential routes of clinical translation in the context of various diseases—both for diagnostic and therapeutic purposes.

## Author contributions

All authors listed have made a substantial, direct and intellectual contribution to the work, and approved it for publication.

### Conflict of interest statement

The authors declare that the research was conducted in the absence of any commercial or financial relationships that could be construed as a potential conflict of interest.

